# Food advertisement influences food decision making and not nutritional status: a study among university students in Ghana

**DOI:** 10.1186/s40795-022-00571-2

**Published:** 2022-08-01

**Authors:** Gabriel Libienuo Sowley Kalog, Faiza Kasim, Bernice Anyebuno, Sandra Tei, Clement Kubreziga Kubuga, Victor Mogre, Paul Armah Aryee

**Affiliations:** 1 Department of Nutritional Sciences, School of Allied Health Sciences, University for Development Studies, Tamale, Ghana; 2 Department of Health Professions Education and Innovative Learning, School of Medicine, University for Development Studies, Tamale, Ghana

**Keywords:** Food advertisement, Food decision making, Body mass index, Students, Internet, Television, Ghana

## Abstract

**Background:**

Consumers are exposed to a wide range of advertisements through different channels daily, which tends to have an influence on their food decision making. The aim of this study was to evaluate the different forms of food advertisements students are exposed to on campus and how they influence their food choices and nutritional status.

**Methods:**

This cross-sectional study was conducted to find out the influence of different forms of food advertisements on students’ food choices and nutritional status. A self-reported semi-structured questionnaire was used to elicit responses from 367 students. About 51.5% of the students were females and 48.5% males. Body Mass index (BMI) was derived from weight and height measured according to standard procedures. Data was analysed and presented as frequencies and percentages. Chi-square was used to determine association between categorical variables (socio-demographic characteristics, food choices and nutritional status).

**Results:**

The students reported ‘use of internet’ (58.9%) as the main source of food advertisement on campus, followed by television (21.0%). A large number of students (74.9%) were affirmative about food advertisements influencing their food decision making. Those with poor nutritional status (underweight, overweight and obese) were more likely to patronize sugar sweetened beverages (10.1%) as compared to fruits and vegetables (1.4%). There was statistical significance (*p* = 0.003) for type of food patronized due to advertisement and the source of advertisement. However, there was no statistical significance (*p* = 0.832) for type of food patronized due to advertisement and BMI of students.

**Conclusion:**

Owing to the increased patronage of internet and television as channels of food advertisements by students, policy makers should prioritize the designing and implementation of intervention programmes through these channels that would influence healthy food decision making and promote consumption of nutrient rich foods. As this population has high self-reported advertisements’ influence on food choices, it is vital to investigate further the influence of contextual cues such as environment and advertisement on their eating habits and dietary patterns.

## Introduction

The choices and intake of processed foods can be induced by factors such as changes in the food environment and variations in the socio-cultural setting [15]. Changes in the food environment include food advertisements and convenience, increased availability and accessibility of processed foods, replacement of traditional diet with Western food, foods as indicators of status. Variations in the socio-cultural setting include long work hours, inactive lifestyles, globalization and urbanization, rise in income levels and decrease in household cooking [15]. Even though the health of an individual is valued as a key driver of such human behaviour, efforts aimed at informing consumers about the relationship between their choice of diets and health in order to influence their eating patterns or food choices have been very challenging [11]. Though the major determinant for eating is hunger, what an individual chooses to eat is not solely driven by physiological or nutritional needs [11].

Food purchasing decisions by consumers are dependent on several factors, therefore there is the need for deeper understanding of these determinants to enhance outcome of successful interventions [11]. Within the food environment there is an increasing spate of advertisement of food which could have varying influences on people. Consumers are exposed to a wide range of advertisement in different media every day, thereby making advertising, sales promotion and public relations essential mass-communication tools available to marketers [2]. Through advertisements, factors such as perceived quality of product, appearance, convenience and cost, greatly determine a consumer’s food decision making [11].

Advertising is a process of communication and every day, consumers are constantly being exposed to a wide range of advertisements from different sources. Thus, advertisements, which serve as a conduit for sales promotion and public relations are vibrant tools available to marketers for mass communication [13]. Advertising is often used to create basic awareness of a product or service in the mind of potential customers in addition to building up knowledge about it [2]. In 2016, almost $13.5 billion was spent on media advertising by more than 20,300 food, beverage, and restaurant companies [18]. Unhealthy food marketing targeting students could be a major contributory factor to poor diet quality and diet related diseases globally [26]. Worldwide, there is an increase in consumption of energy-dense foods that are high in fat, salt and sugars, but poor in vitamins, minerals and other micronutrients as well as dietary fibre [25]. For majority of students who mostly live away from their families/homes and have to make independent food choices during periods that the university is in session, food adverts could have a great influence on their lives [4]. Unhealthy food selection, increased cost of healthy foods and the ease of availability of fast foods at university campuses, could negatively impact on students eating behaviours [9].

There are many products and services including food products which are presented to consumers and potential consumers via advertisement [2]. Vigorous promotional practices through television advertising could have contributed significantly to the erosion of diet quality among many cultures [10]. Some studies have found out that, portion size, the behaviour of nearby eaters, the accessibility of food and even dubious health claims through advertisements all affect the amount and type of food people purchase or consume [6, 23]. Studies in recent times have shown that foods of low nutritional value are often greatly marketed in low-income and marginalized neighbourhoods [16, 19]. All over the world, people are routinely being exposed to advertising and marketing through radio, television, magazines, internet (which includes social media and other web-based applications), schools, product placements, cell phones, video games as well as other means [3]. These advertisements and marketing strategies are purposefully designed to increase brand recognition, loyalty and quite sadly sales of high calorie and unhealthy foods. Most of these advertised products contain excess amounts of saturated fats, added sugar, and salts and, at the same time, do not promote adequate intakes of fruits, vegetables and whole grains [3].

Food choices and intake are important factors that can influence the weight and overall nutritional and health status of an individual [7]. Thus, it becomes imperative to investigate the link between adverts that may influence such behaviours and their various outcomes. Even though there is empirical evidence to show that food advertisement has influence on food choices of people of all age groups [2], little is known about the connection between these variables among university students in Ghana. In view of this dearth in literature, this study aimed to differentiate forms of food advertisement students are exposed to on campus and how these influence their food choices and nutritional status.

## Materials and methods

### Study area and design

This study was conducted on the Tamale campus of the University for Development Studies (UDS) in the Northern Region of Ghana. There are a total of seven (7) schools/faculties with several undergraduate and postgraduate programmes being ran at the Tamale campus. A cross-sectional study design was adopted in this study.

### Study population and sampling

The sample size of the study was determined using the formula: *n* = (X^2^NP (1-P)) ÷ (e^2^ (N-1) + X^2^P (1-P)). Where *n* = required sample size, X^2^ = the table value of chi-square for 1 degree of freedom at the desired confidence level (3.841), *N* = population size (8000), *P* = population proportion (assumed to be 0.50, to provide maximum sample size), and e = degree of accuracy expressed as a proportion (0.05) [14]. Substituting the values into the formula gave a sample size of 367. Thus, 367 students were selected from total enrolled in six faculties/schools at the Tamale campus of the UDS. A sample proportionate to the student population in each faculty was drawn. Each faculty/school was visited during the period of data collection (from 6^th^—17^th^ September, 2021) and all students present in randomly selected lecture halls, were given equal opportunity to participate in the study by writing “yes” and “no” on sheets of paper which were shuffled for students to pick. All students who picked “yes” were included for the study, this was repeated until the desired sample size was achieved for each of the school/faculty. The general student population at the Tamale campus as at the time of the study was about 8000.

### Data collection methods

A questionnaire, specifically designed to evaluate the different forms of food advertisements students are exposed to on campus and how they influence their food decision making and nutritional status, consisted of 28 items. The items of the questionnaire were reviewed for content validity by a team of nutritionists, behavioural scientists and public health specialists.

In order to ensure the reliability of the study findings, pre-testing was conducted to 20 subjects previously to test the suitability of the questionnaire. The pre-testing helped to ensure that the items are meaningful to the target population and minimises subsequent measurement errors. The questionnaire was hand-delivered to each selected respondent after briefing them on how to respond to the various items and seeking their consent. The questionnaire was self-administered, with the anthropometric assessments undertaken by a team of final year nutrition students. Respondents were offered the opportunity to ask questions on any issue which they did not understand and clarifications were provided. Each student completed their questionnaire within ten (10) to fifteen (15) minutes and returned it to a team of data collectors. Non-response rate was zero percent. The questionnaire was used to collect data on socio-demographic characteristics of respondents, sources of food advertisement and the influence of food advertisement on students’ food decision making.

### Anthropometric assessment

Measurements of weight and height for each student was done following World Health Organization (WHO) standard procedures [27]. The weights and heights of respondents were measured after they had submitted their completed questionnaires. Students were weighed in light clothing and without shoes, using a Seca digital flat scale, to the nearest 0.1 kg. Their heights were measured to the nearest 0.1 cm using a standardized Seca stadiometer. The weights and heights were used to determine the body mass index (BMI) of participants based on the calculation weight/height^2^ (kg/m^2^).

### Data analysis

The data was analysed using IBM SPSS for Windows version 20. Categorical variables have been presented as frequencies and percentages. To examine associations between socio-demographic characteristics, food habits and the nutritional status, Chi-Square test was performed. Fischer’s exact test was used in cases where conditions for Chi-Square test were not met. *P*-value of < 0.05 was considered significant at 95% confidence interval.

## Results

Overall, there were 367 respondents for the study, out of which 189 (51.5%) were females. The mean age of respondents was 23.14 years with majority (52.9%) falling within the age group of 23–27 years. The least (2.2%) respondents were in the age group of 28–32 years. Most (64.0%) of the respondents who participated in the study said they were Christians, whilst a good proportion (30.2%) associated themselves with the Islamic religion. Students in level 400 (Table 1) constituted majority (30.0%) of the respondents. Also, a majority of the respondents (47.4%) indicated that they received a monthly income within a range of 400 to 600 (GHS). A good proportion (26.7%) of students indicated that their monthly incomes were within the range of 100 – 300 GHS. There were a few students (3.5%) who received 1000 GHS and above as monthly income. Table 1 shows the details of the socio-demographic characteristics of the study participants.

**Table 1:** Respondents socio-demographic characteristics

Variable	Frequency	Percentage (%)
	**(*n* = 367)**	
**Age (in years)**		
< 18	11	3.0
18 – 22	144	39.2
23 – 27	194	52.9
28 – 32	8	2.2
33 +	10	2.7
**Gender**		
Male	178	48.5
Female	189	51.5
**Religion**		
Christianity	235	64.0
Islam	111	30.2
African Traditional Religion	19	5.2
Other	2	0.6
**Level at the University**		
100	61	16.6
200	87	23.7
300	71	19.3
400	110	30.0
^a^Other	38	10.4
**Monthly Income (in GHS)**		
< 100	22	6.0
100—300	98	26.7
400—600	174	47.4
700—900	60	16.4
1000 +	13	3.5

^a^students of level 500 and 600

**Fig. 1:**
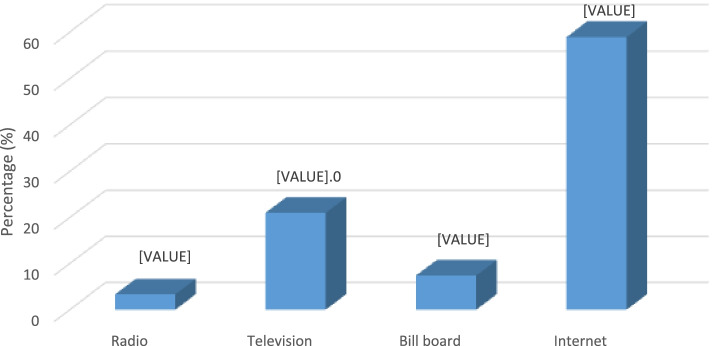
Respondents source of food advertisement on university campus

Respondents were asked whether food advertisement influenced their food selection, and majority (74.9%) of them answered in the affirmative. About 91% of students said they have seen or heard about food advertisements on campus. Among the students who had seen or heard about food advertisements on campus, majority (58.9%) said their source was through the internet (including social media). A good proportion (21.0%) of the students cited television as their source of food advertisement, whilst radio was the least indicated (3.3%). Figure 1 shows the details on respondents’ sources of food advertisement on the university campus.

Among the factors which influenced respondents’ food choices, the appearance of food was of greater influence (31.9%). Concerning the aspect of food advertisement which influenced their food choice, about 43.6% of respondents indicated that ‘taste’ of advertised foods was more likely to influence their food decision making. It is worth noting that irrespective of advertisement, ‘taste’ had much influence on the food choices of respondents. Majority (44.7%) of students were found to have patronized advertised foods monthly, followed by weekly patronage of advertised foods which was reported by about 32.4% of students. Patronage of sugar sweetened beverages due to advertisement was also reported among most (36.0%) of the study participants, whilst patronage of fruits and vegetables due to advertisement was found to be lowest (5.4%) among respondents. Regarding the level of importance of food advertisement, about 49.3% of the students reported that it was important to them, with an appreciable proportion (27.0%) indicating food advertisement as very important. The details on factors of food and aspects of food advertisement which influenced respondents’ food choices are shown in Table 2.

**Table 2:** Factors of food and its advertisement which influence respondents’ food choice

Variable	Frequency	Percentage (%)
	**(*n* = 367)**	
**Factors which influenced respondents food choices without advertisement**		
Name and familiarity with food	94	25.6
Cooking method	77	21.0
Appearance	117	31.9
Taste	79	21.5
**Factors which influenced respondents food choices with advertisement**		
Brand	90	24.5
Price	117	31.9
Taste	160	43.6
**Patronage of advertised foods**		
Daily	63	22.9
Weekly	89	32.4
Monthly	123	44.7
**Type of food patronized due to adverts**		
Beverages (sugar sweetened)	132	36.0
Pastries (high fat)	106	28.9
Meals	109	29.7
Fruits and Vegetables	20	5.4
**Level of importance of food adverts**		
Not important	87	23.7
Important	181	49.3
Very important	99	27.0

The study looked at how respondents’ socio-demographic characteristics influenced patronage of advertised foods, type of food patronized due to advertisement and level of importance of food advertisement. The findings revealed that a good proportion (30.2%) of students within the age group of 23–27 years, were more likely to patronize advertised foods weekly followed by about 20.7% of students in the age group of 18–22 years. Daily patronage of advertised foods was reported among 13.1% of students in the age group of 23–27 years. It is important to note that daily, weekly and monthly patronage of advertised foods was noticed among respondents in the age group of 23–27 years. However, these relationships were not statistically significant (*p* = 0.986).

Furthermore, students in the age group of 23–27 years were most likely to patronise advertised sugar sweetened beverages (19.6%), high fat pastries (16.3%) and local meals/dishes (14.2%). About 13.9% of students in the age group of 18–22 years also reported they patronized sugar sweetened beverages due to advertisement. Patronage of fruits and vegetables was found to be low among students of all age groups. These relationships were also not statistically significant (*p* = 0.056).

Considering the level of importance of food adverts, about 27.8% and 18.0% of students in the age groups of 23–27 and 18–22 years respectively, considered them to be important. Whilst about 14.4% of students also within the age group of 23–27 years reported that food advertisement was very important to them in their food decision making.

In terms of gender, the findings from this study showed that daily patronage of advertised foods was more likely to occur among females (14.0%) than males (11.2%). Also, patronage of sugar sweetened beverages (20.4%) and high fat pastries (15.3%) due to advertisement was found to be high among female students than male students. However, more male students (15.8%) patronized local meals/dishes due to advertisement compared to their female counterparts (13.9%). Even though patronage of fruits and vegetables was generally low among both gender, more females (3.5%) patronized fruits and vegetables due to advertisement compared to males (1.9%). There were more female students (26.4%) who indicated that food advertisement was important in their food decision making than male students (22.9%).

It was further revealed that majority (26.4%) of students whose monthly income ranged between 400 – 600 GHS, were more likely to patronize advertised foods weekly. A good proportion (14.0%) whose monthly income was within a range of 100 – 300 GHS also patronized advertised foods weekly. About 12.3% of the students whose monthly income ranged between 400 – 600 GHS indicated that they patronized advertised foods daily. It is important to note that, students with income levels within the range of 400 – 600 GHS were more likely to patronized advertised foods daily, weekly and monthly. There was no statistical significance (*p* = 0.317) for monthly income of respondents and frequency of patronage of advertised foods.

Majority (20.0%) of students whose monthly income was within a range of 400 – 600 GHS spent their monies on sugar sweetened beverages. About 14.2% of students whose monthly income was in same range spent their monies on meals/dishes (i.e. *banku/kenkey* with soup or stew, *waakye/*rice with stew or soup, *fufu/kokonte* with soup, *tuo zaafi* with soup etc.). There was usually meat, fish or egg included in all local meals/dishes served. Patronage of fruits and vegetables was low across all monthly income levels among respondents. There was statistical significance (*p* = 0.001) for students’ monthly income and the type of food patronized as a result of advertisement.

A greater percentage (25.9%) of students whose monthly income was within the range of 400 – 600 GHS said food advertisement was important in their food decision making. A significant proportion (13.0%) with monthly income in the range of 100 – 300 GHS also reported that food advertisement was important in their food decision making. A good proportion (12.0%) with monthly income in the range of 400 – 600 GHS reported food advertisement as very important in their food decision making. There was no statistical significance (*p* = 0.053) for monthly income of students and level of importance of food advertisement.

Students of level 400 at the university were more likely to patronize advertised foods daily (7.6%), weekly (15.8%) and monthly (6.5%). Patronage of sugar sweetened beverages was found to be more (10.9%) among level 200 students, followed by students of level 400 (10.4%). Patronage of high fat pastries was found to be more among level 400 students (9.0%) and level 300 students (7.1%). Food advertisement was considered to be important (15.8%) and very important (7.6%) in the food decision making of level 400 students. There was no statistical significance (*p* = 0.316) for students’ level at the university and the level of importance of food advertisement. The details are shown in Table 3.

**Table 3:** How respondents socio-demographic characteristics influence patronage of advertised foods, type of food patronised due to advertisement and level of importance of food advertisement

Variable	Patronage of advertised foods			*p*—value	Type of food patronized due to adverts				*p*—value	Level of importance of food adverts			*p*—value
	**Daily**	**Weekly**	**Monthly**		**Beverages**	**Pastries**	**Meal**	**Fruits and Vegetables**		**Not Important**	**Important**	**Very Important**	
	**n (%)**	**n (%)**	**n (%)**		**n (%)**	**n (%)**	**n (%)**	**n (%)**		**n (%)**	**n (%)**	**n (%)**	
**Age (in years)**													
< 18	2 (0.5)	6 (1.6)	3 (0.8)		3 (0.8)	4 (1.1)	4 (1.1)	0 (0.0)		2 (0.5)	7 (2.0)	2 (0.5)	
18—22	37 (10.1)	76 (20.7)	31 (8.5)		51 (13.9)	39 (10.6)	47 (12.8)	7 (1.9)		39 (10.6)	66 (18.0)	39 (10.6)	
23—27	48 (13.1)	111 (30.2)	35 (9.5)		72 (19.6)	60 (16.3)	52 (14.2)	10 (2.7)		39 (10.6)	102 (27.8)	53 (14.4)	
28—32	2 (0.5)	5 (1.4)	1 (0.3)		1 (0.3)	3 (0.8)	4 (1.1)	0 (0.0)		4 (1.1)	2 (0.5)	2 (0.5)	
33 +	3 (0.8)	5 (1.4)	2 (0.5)	0.986	5 (1.4)	0 (0.0)	2 (0.5)	3 (0.8)	0.056	3 (0.8)	4 (1.1)	3 (0.8)	0.522
**Gender**													
Male	41 (11.2)	101 (27.5)	30 (8.2)		57 (15.5)	50 (13.6)	58 (15.8)	7 (1.9)		39 (10.6)	84 (22.9)	49 (13.4)	
Female	51 (14.0)	102 (27.8)	42 (11.4)	0.436	75 (20.4)	56 (15.3)	51 (13.9)	13 (3.5)	0.306	48 (13.1)	97 (26.4)	50 (13.6)	0.804
**Monthly Income (in GHS)**													
< 100	5 (1.4)	11 (3.0)	6 (1.6)		10 (2.7)	8 (2.2)	3 (0.8)	1 (0.3)		10 (2.7)	6 (1.6)	6 (1.6)	
100—300	22 (6.0)	51 (14.0)	25 (6.8)		21 (5.7)	32 (8.7)	36 (9.8)	9 (2.5)		25 (6.8)	47 (13.0)	26 (7.1)	
400—600	45 (12.3)	97 (26.4)	32 (8.7)		73 (20.0)	42 (11.4)	52 (14.2)	7 (1.9)		35 (9.5)	95 (25.9)	44 (12.0)	
700—900	14 (3.8)	39 (10.6)	7 (1.9)		26 (7.1)	21 (5.7)	13 (3.5)	0 (0.0)		12 (3.3)	31 (8.4)	17 (4.6)	
1000 +	6 (1.6)	5 (1.4)	2 (0.5)	0.317	2 (0.5)	3 (0.8)	5 (1.4)	3 (0.8)	0.001	5 (1.4)	2 (0.5)	6 (1.6)	0.053
**Level at the University**													
100	22 (6.0)	27 (7.4)	12 (3.3)		18 (4.9)	19 (5.2)	20 (5.4)	4 (1.1)		10 (2.7)	33 (9.0)	18 (4.9)	
200	20 (5.5)	46 (12.5)	21 (5.7)		40 (10.9)	18 (4.9)	25 (6.8)	4 (1.1)		30 (8.2)	35 (9.5)	22 (6.0)	
300	14 (3.8)	44 (12.0)	13 (3.5)		22 (6.0)	26 (7.1)	20 (5.4)	3 (0.8)		15 (4.1)	34 (9.3)	22 (6.0)	
400	28 (7.6)	58 (15.8)	24 (6.5)		38 (10.4)	33 (9.0)	33 (9.0)	6 (1.6)		24 (6.5)	58 (15.8)	28 (7.6)	
Other	8 (2.2)	28 (7.6)	2 (0.5)	0.086	14 (3.8)	10 (2.7)	11 (3.0)	3 (0.8)	0.738	8 (2.2)	21 (5.7)	9 (2.5)	0.316

In looking at how food advertisement influenced patronage of advertised foods on the university campus, it was revealed that majority (36.1%) of students whose source of food advertisement was through the internet, patronized advertised foods weekly, whilst about 17.2% patronized advertised foods daily and 11.7% monthly. This was followed by students for whom television was their source of food advertisement, about 14.5% of them patronised advertised foods weekly. There was no statistical significance (*p* = 0.248) for source of food advertisement and patronage of advertised foods among students.

For the aspect of advertised foods which influenced respondents’ food choice, most (28.0%) of the respondents indicated they patronised advertised foods monthly due to the taste of the food. There was a good percentage (16.7%) of respondents for whom the price of advertised foods influenced their patronage monthly. The brand of advertised foods also influenced patronage among 11.3% of the respondents monthly. Weekly patronage of advertised foods due to the brand was recorded as the least (5.1%) among the students on the university campus. There was no statistical significance (*p* = 0.312) for the different aspects of advertised foods and their patronage at the time of the study. Table 4 shows the details.

**Table 4:** How food advertisement influence Patronage of advertised foods

Variable	Patronage of advertised foods			
	**Daily**	**Weekly**	**Monthly**	***p*-value**
	**n (%)**	**n (%)**	**n (%)**	
**Source of Food Advertisement**				
Radio	4 (1.2)	7 (2.1)	1 (0.3)	
Television	12 (3.6)	48 (14.5)	17 (5.1)	
Bill board	8 (2.4)	11 (3.3)	8 (2.4)	
Internet	57 (17.2)	120 (36.1)	39 (11.7)	0.248
**Aspect of Advert’s influence on food choice**				
Brand	18 (6.5)	14 (5.1)	31 (11.3)	
Price	25 (9.1)	18 (6.5)	46 (16.7)	
Taste	30 (10.9)	16 (5.8)	77 (28.0)	0.312

The study examined whether there was any association between BMI of respondents and aspects of food advertisements. The findings showed that weekly patronage of advertised foods was reported more among participants with BMI of all categories. An appreciable proportion of students (12.8%) who were found to be overweight/obese, patronized advertised food weekly. Daily, weekly and monthly patronage of advertised foods was found to be low among respondents who were underweight (2.4%) as compared to the other BMI classifications. There was no statistical significance (*p* = 0.909) for patronage of advertised foods and the BMI classification of students.

Overall, patronage of advertised sugar sweetened beverages was high (36.0%) among respondents across all BMI classifications. This was followed by patronage of local meals/dishes (29.7%) and high fat pastries (28.8%). For respondents who were overweight/obese, patronage of sugar sweetened beverages and high fat pastries was reported among 9.0% and 7.0% respectively. Patronage of fruits and vegetables among study population was low across all BMI classifications. There was no statistical significance (*p* = 0.832) for BMI classification and type of food patronized as a result of advertisement among participants.

The study also revealed that about 11.4% of students who were overweight/obese reported that food advertisement was important to them, whilst about 6.3% of respondents with same BMI classification considered food advertisement to be very important to them. There was no statistical significance (*p* = 0.756) for level of importance of food advertisement and BMI classification of respondents.

The internet was reported as the main source of food advertisement for a good proportion (15.7%) of students who were overweight/obese. Television was also found to be a source of food advertisement for about 5.7% of respondents who were also overweight/obese. The details are shown in Table 5.

**Table 5:** Aspects of food advertisement and their influence on respondents BMI

Variable	BMI Classification				
	**Underweight**	**Normal**	**Overweight**	**Obese**	
	**n (%)**	**n (%)**	**n (%)**	**n (%)**	***p*—value**
**Patronage of advertised foods**					
Daily	1 (0.3)	71 (19.3)	17 (4.6)	3 (0.8)	
Weekly	6 (1.6)	150 (40.9)	40 (10.9)	7 (1.9)	
Monthly	2 (0.5)	53 (14.4)	16 (4.4)	1 (0.3)	0.909
**Type of food patronized**					
Beverages	4 (1.1)	95 (25.9)	28 (7.6)	5 (1.4)	
Pastries	1 (0.3)	79 (21.5)	24 (6.5)	2 (0.5)	
Local meals	3 (0.8)	85 (23.2)	17 (4.6)	4 (1.1)	
Fruits and Vegetables	1 (0.3)	15 (4.1)	4 (1.1)	0 (0.0)	0.832
**Level of importance of food adverts**					
Not important	3 (0.8)	65 (17.7)	16 (4.4)	3 (0.8)	
Important	2 (0.6)	137 (37.3)	36 (9.8)	6 (1.6)	
Very important	4 (1.1)	72 (19.6)	21 (5.7)	2 (0.6)	0.756
**Source of food advertisement**					
Radio	0 (0.0)	11 (3.3)	1 (0.3)	0 (0.0)	
Television	1 (0.3)	57 (17.2)	18 (5.4)	1 (0.3)	
Bill board	1 (0.3)	22 (6.6)	3 (0.9)	1 (0.3)	
Internet	6 (1.8)	158 (47.6)	43 (13.0)	9 (2.7)	0.753

BMI classification [24]: BMI < 18.5 kg/m^2^ (underweight), 18.5 kg/m^2^ – 24.9 kg/m^2^ (normal) 25 kg/m^2^ – 29.9 kg/m^2^ (overweight), ≥ 30 kg/m^2^ (obese)

The findings from the study revealed that majority (24.7%) of students’ patronized local meals/dishes advertised through the internet. About 22.9% and 14.2% of the respondents said they patronized sugar sweetened beverages and high fat pastries respectively, which were also advertised through the internet. High fat pastries were also patronized by about 9.9% of students, due to advertisement through television. There was low patronage of fruits and vegetables (5.1%) across all sources of food advertisement on the university campus. There was statistical significance (*p* = 0.003) for the source of food advertisement and the type of food patronized as a result of advertisement. Table 6 below shows the details.

**Table 6:** How respondents’ source of food advertisement influence type of food patronized

Variable	Type of food patronized				
	**Beverages**	**Pastries**	**Local meal**	**Fruits and Vegetables**	***p*-value**
	**n (%)**	**n (%)**	**n (%)**	**n (%)**	
**Source of food advert**					
Radio	4 (1.2)	6 (1.8)	1 (0.3)	1 (0.3)	
Television	30 (9.0)	33 (9.9)	11 (3.3)	3 (0.9)	
Bill board	7 (2.1)	9 (2.7)	9 (2.7)	2 (0.6)	
Internet	76 (22.9)	47 (14.2)	82 (24.7)	11 (3.3)	0.003

The study also sought to find out the level of importance of food advertisement and aspects of the advertisement which influenced choice of food among participants. Table 6 shows that for students who considered food advertisement to be important, majority (26.9%) of their food choices were influenced by the taste and followed by about 20.4%, whose food choices were influenced by the prices. For students who considered food advertisement to be very important, about 11.6% each of them regarded the taste and brand as aspects that would influenced their food choices. There was statistical significance (*p* ˂ 0.001) for the level of importance of food advertisement and the aspect of advertisement’s influence on respondents’ food choices. Table 7 shows the details.

**Table 7:** Level of importance of food advertisement and aspect of advert’s influence on respondents’ food choice

Variable	Level of importance of food advertisement			
	**Not Important**	**Important**	**Very Important**	***p*-value**
	**n (%)**	**n (%)**	**n (%)**	
**Aspect of Advert’s influence on food choice**				
Brand	5 (1.8)	26 (9.5)	32 (11.6)	
Price	18 (6.5)	56 (20.4)	15 (5.5)	
Taste	17 (6.2)	74 (26.9)	32 (11.6)	< 0.001

## Discussion

This study showed that internet was the source of food advertisement to a greater proportion (58.9%) of students on the university campus. This was followed by television (21.0%), billboards (7.4%) and radio (3.3%). Television viewing, convenience stores and the internet have become the most popular sites for young people to be exposed to food advertising [17]. The implication of this finding in this study is that appropriate health authorities could take advantage of the increased patronage of the internet and television as channels to design effective nutrition education programmes targeting students at the level of tertiary institutions.

Aside the cost of food products, other factors such as perceived quality, convenience and appearance influence the decision making of consumers at supermarkets and shopping centres [21]. The findings from this research are similar, preference to appearance, name/familiarity and taste of food were notably discovered in this study to be influential in determining the food choices among most of the students on the campus environment. Appearance and taste are sensory aspects of food which are thought to influence spontaneous choices of food. Additionally, the taste of advertised foods was revealed to have influenced the food decision making of majority (43.6%) of the respondents. In addition, most of the students who considered food advertisement as important, had their food choices influenced by taste (26.9%) and price (20.4%). These findings were found to be in line with other studies which presented similar findings; for example, [12] suggested that, in addition to social and cultural factors, taste preference and past food habits or familiarity with food contributed significantly to food choices among students. The implication of the findings in this current study is that food advertising practitioners would need to pay more attention to the taste and appearance when working on adverts targeting students at tertiary institutions. This study also revealed that the choice of food products by majority (74.9%) of participants was influenced by advertisement. This is slightly higher when compared to a study on “food choice behaviours among Ghanaians” in Accra, which findings showed that 44.1% of food choices among respondents’ was influenced by advertisement [11].

In this study, the prevalence of underweight (2.5%), overweight (19.9%) and obesity (3.0%) were similar to findings from a study on dietary habits and nutritional status of medical students in three state universities in Cameroon where 4.9%, 21.7% and 3.0% of the students were found to be underweight, overweight and obese respectively [5]. The findings in this study implied that using BMI as an indicator of nutritional status, about 25.4% of the students were malnourished.

The study also revealed less patronage of fruits and vegetables (5.4%) due to advertisement among students, whilst more students (36.0%) were found to patronize sugar sweetened beverages due to advertisement. Fruits and vegetables intake in this study are comparably lower than findings from other studies. Whilst about 71.0% of all respondents had eaten at least, a fruit or vegetable the previous day; there were 67.0% of students from University of Florida compared to 57.0% of students from Arkansas State University that had consumed at least a fruit or vegetable [22]. However, the low fruits and vegetables consumption among students in this study is in line with findings of another study by Freedman, which found that first year students who relocated to campus decreased their intake of fruits, vegetables and dairy as well as meal frequency [8]. Intake of fruits and vegetables is one of the important healthy behaviours to achieve an individual’s optimum physical function [1]. Frequent food and beverage patronage around campus was found to be associated with reduced frequency of breakfast consumption and high fat and added sugar intake [20]. Thus, it is important for nutrition policy makers in Ghana to develop interventions tailored at university students to promote consumption of fruits and vegetables.

The strength of this study is that, it has provided some insight into the nature of food advertisement on the university campus and how students’ food choices are being influenced by advertisement. One of the limitations of the study is that, it was conducted at the Tamale campus of the University for Development Studies. Future studies could be extended beyond the University for Development Studies to include more universities in the country. Also, the study relied on the report from respondents, which could be subjected to recall bias and social desirability. Again, being a cross-sectional study, it was not possible to establish causality.

## Conclusion

The food decision making among students at the university campus was found to be influenced by factors such as advertisement, taste, price, familiarity and appearance. The dominant source of food advertisement on the campus of the University for Development Studies was found to be through the internet. Television was also revealed as an important source of food advertisement to students on campus. Nutritional status, using BMI as an indicator, was not influenced by food advertisement. Also, patronage of advertised fruits and vegetables among students on the university campus was found to be low. However, there was increased patronage of sugar sweetened beverages, meals/dishes and high fat pastries among students.

Therefore, appropriate health authorities should take advantage of the increased patronage of internet and television as key sources of food advertisement on the university campus to effectively plan and design nutritional intervention programmes with the aim to improve food decision making and promote consumption of nutritious foods for good health among students. Additionally, as this population has high self-reported advertisements’ influence on food choices, it is vital to investigate further the influence of contextual cues such as environment and advertisement on their eating habits and dietary patterns.

## Data Availability

The datasets which was generated and analysed during the current study and used for the preparation of the manuscript are included in the article submitted for publication.

## Abbreviations

BMI: Body Mass Index
UDS: University for Development Studies
SPSS: Statistical Package for Social Sciences
WHO: World Health Organization
